# Supplementary material for: Revolutionising Agricultural Sustainability: New ‘Furrow Tillage’ can Mitigate Short‐Term Soil‐to‐Atmosphere CO2 Flux and Promote Soil‐Plant‐Microbe Health
Source: Adv Sci (Weinh). 2026 Jul 17:e76645. Online ahead of print. doi: 10.1002/advs.76645 (PMC13379218; doi:10.1002/advs.76645)
Supplement: Supplementary file 1 — Supporting File: advs76645‐sup‐0001‐SuppMat.docx. [file ADVS-9999-e76645-s001.docx]

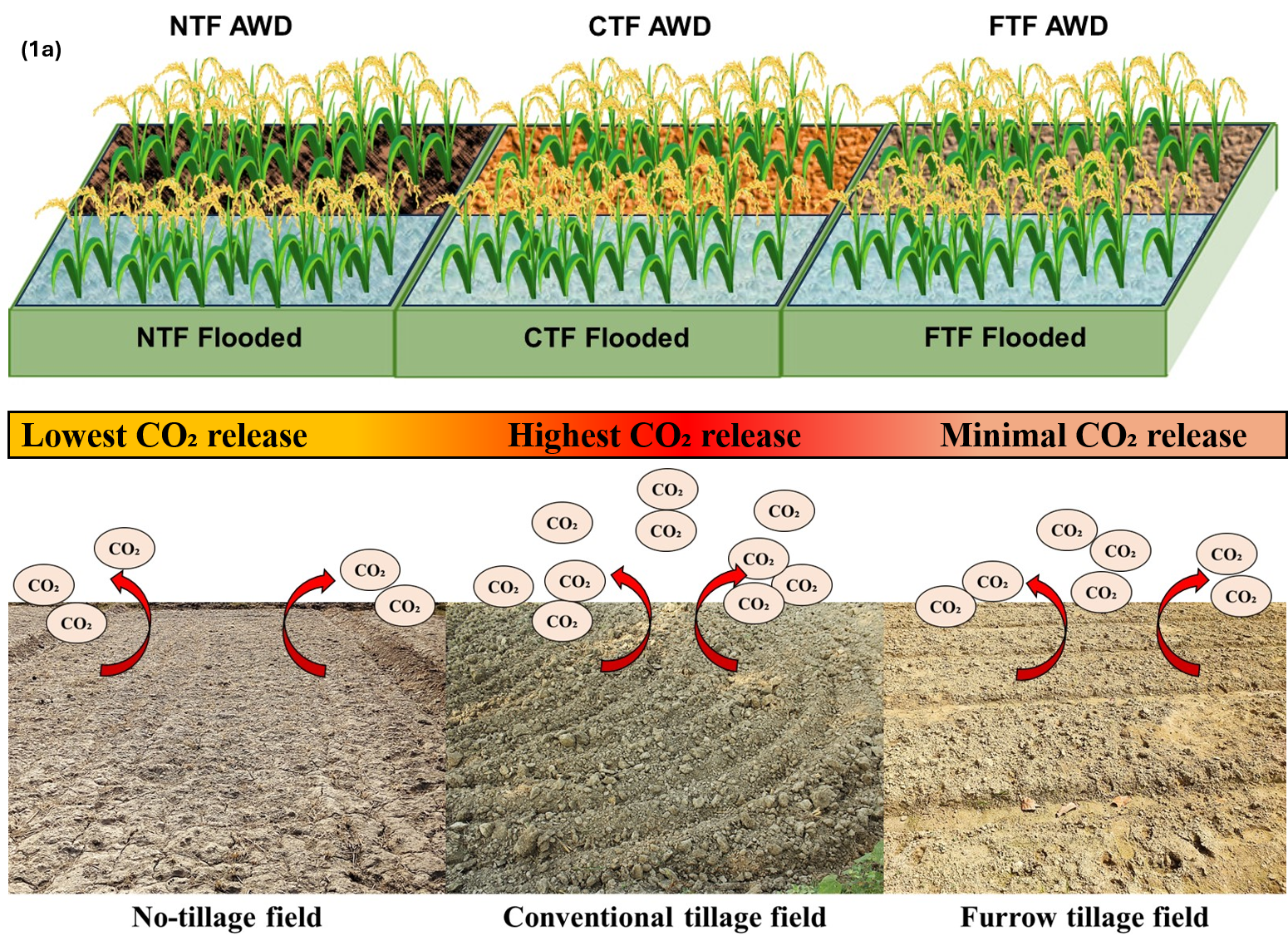


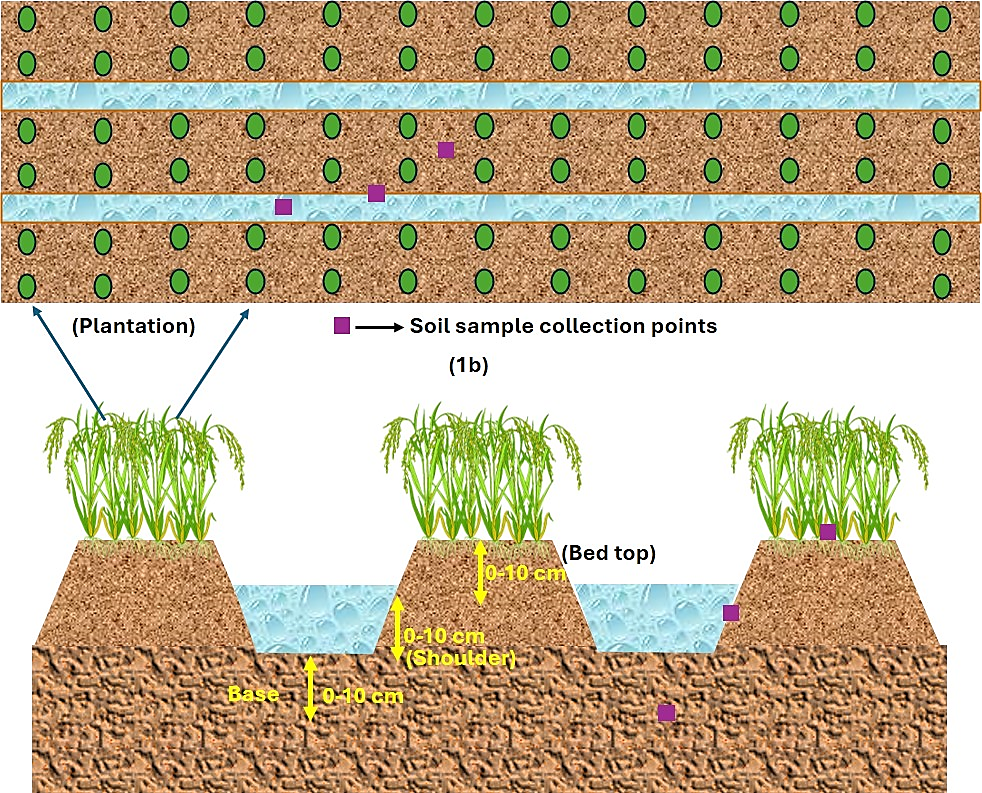


**Supplementary figure 1.** The three field setups are presented in a combination of schematic diagrams of irrigation regimes and varied tillage practices in the field (**a**). Released carbon dioxide (CO_2_) bar is a colored illustration of analysed data during sampling phases within the two-year span. Soil sample collection points are presented (**b**) in this graphic from three spots at each site.


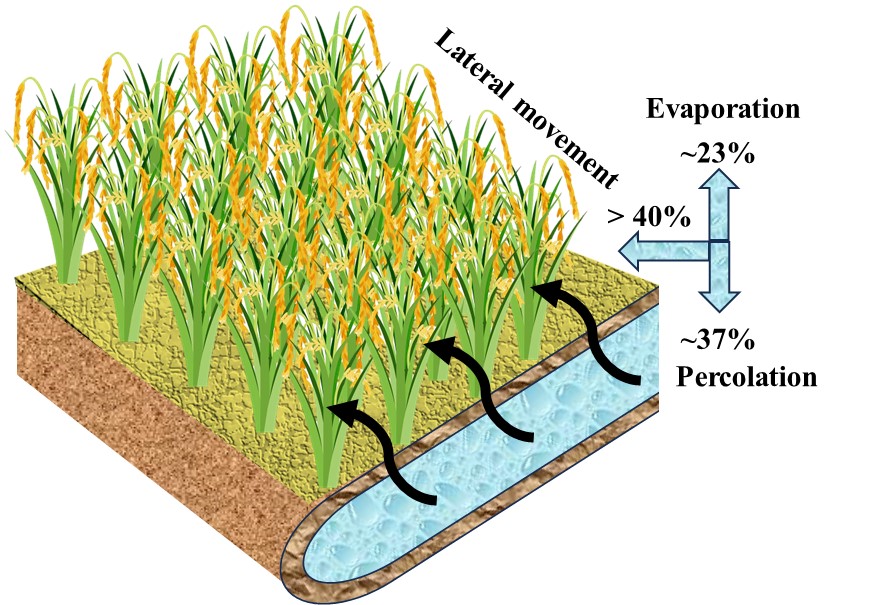


**Supplementary figure 2.** Furrow tillage water movement in three directions, as per the soil clods and water passage modelling.


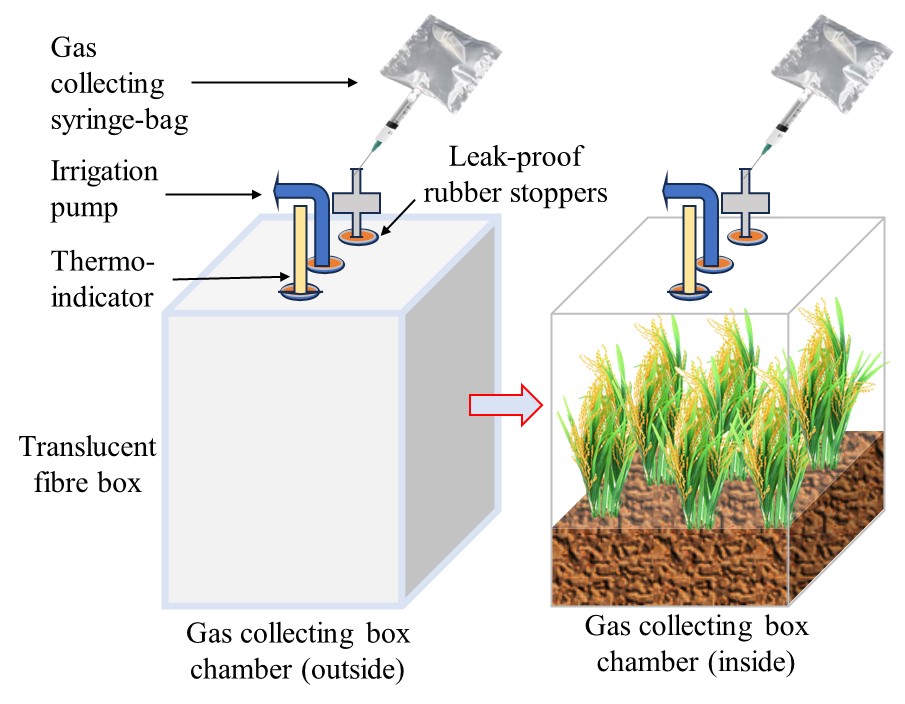


**Supplementary figure 3.** Schematic of a carbon dioxide gas collection chamber setup in the field.


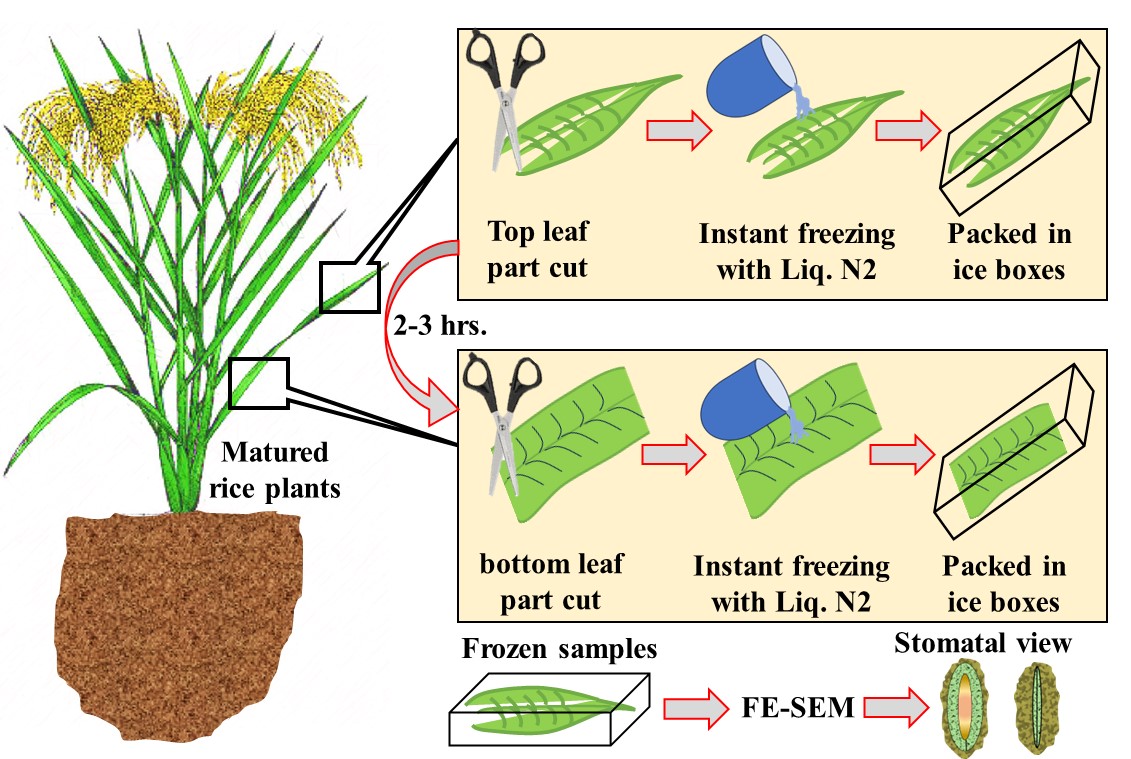


**Supplementary figure 4.** Plant leaf sampling and snap-freezing for internal structural observation.


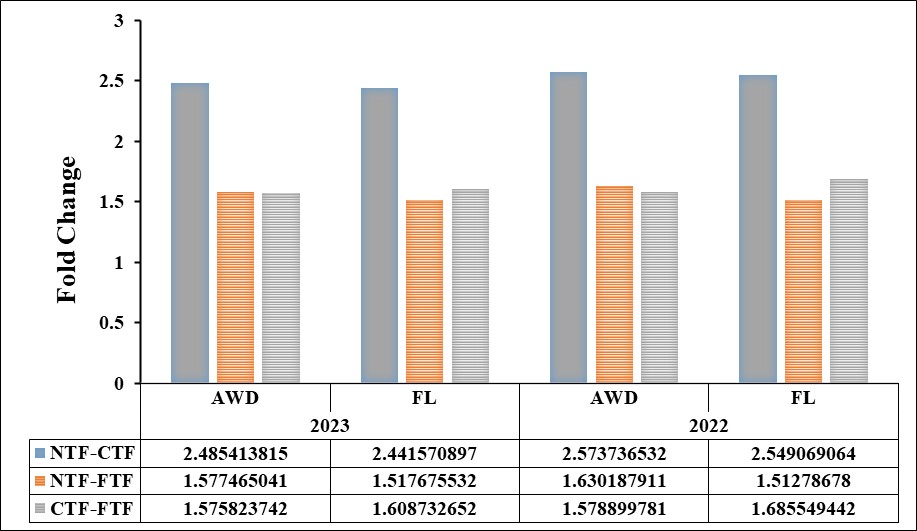


**Supplementary figure 5.** Fold change of released CO_2_ from three field setups in two consecutive years. The data represents the average trend results from the 12 experimental sites. This data has been further justified by one-way ANOVA at p<0.05 significance level.


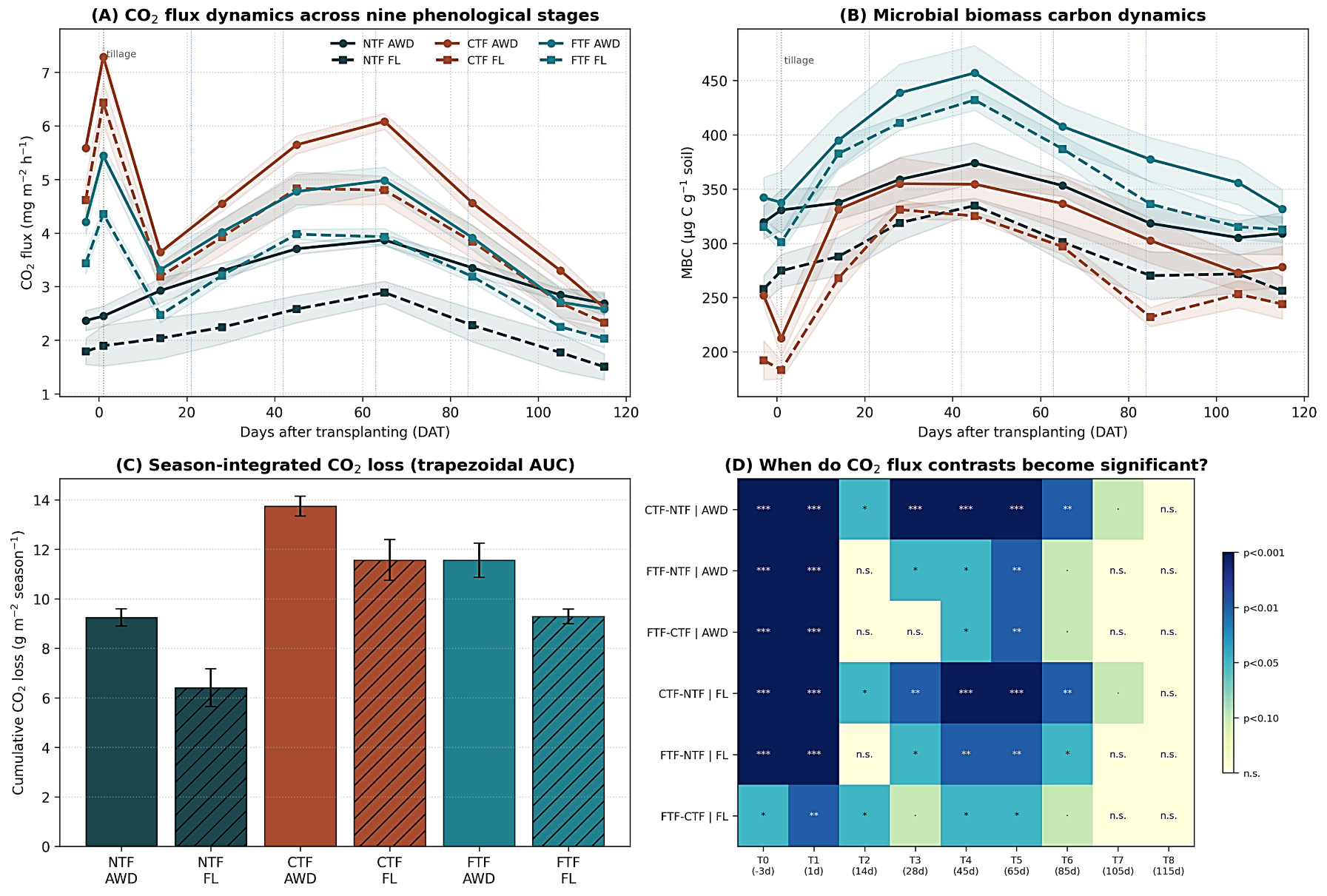


**Supplementary figure 6.** Temporal carbon dynamics across the setups and seasons (cropping cycles).


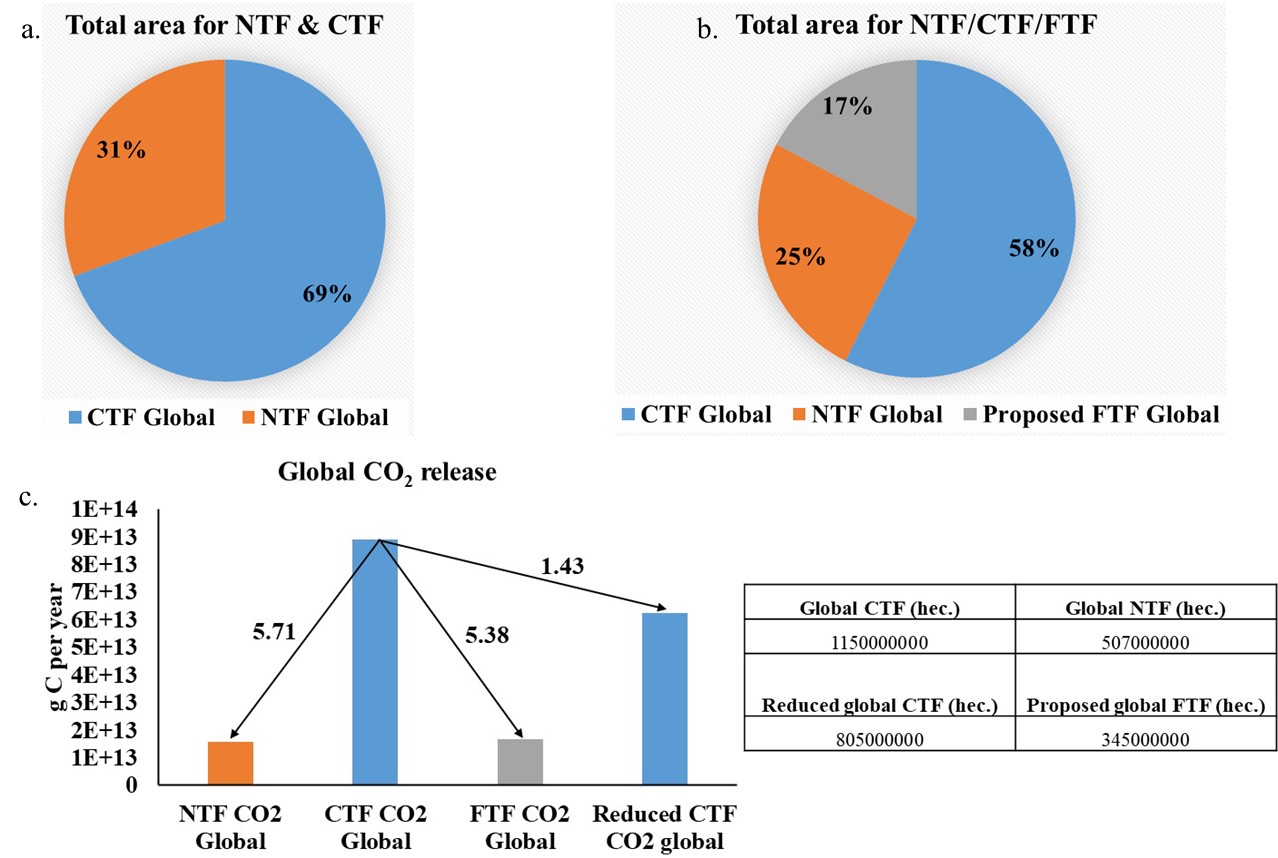


**Supplementary figure 7**. Global agronomic field area for NTF and CTF with proposed FTF area coverage(a-b), and fold change in CO_2_ release if FTF is followed globally (c).


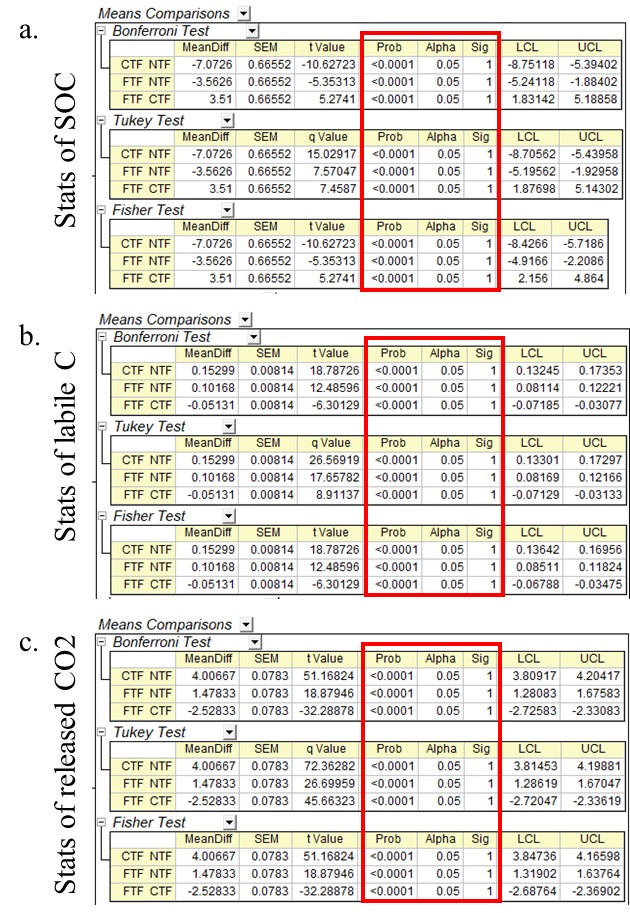


**Supplementary figure 8**. Post-hoc statistical analyses after one-way ANOVA of SOC, labile carbon content and released CO_2_ from fields using Bonferroni correction, Tukey HSD, and Fisher LSD tests. The red-lined box marked scores of 1, indicating statistical significance.


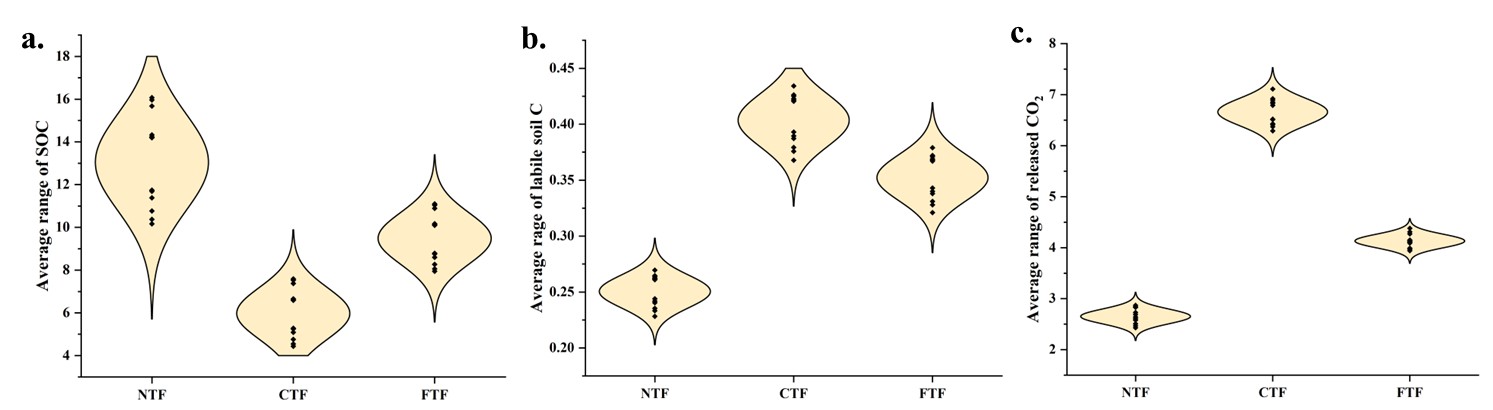


**Supplementary figure 9**. Average data trend of SOC, labile soil carbon and released CO_2_ in the normal distribution model.


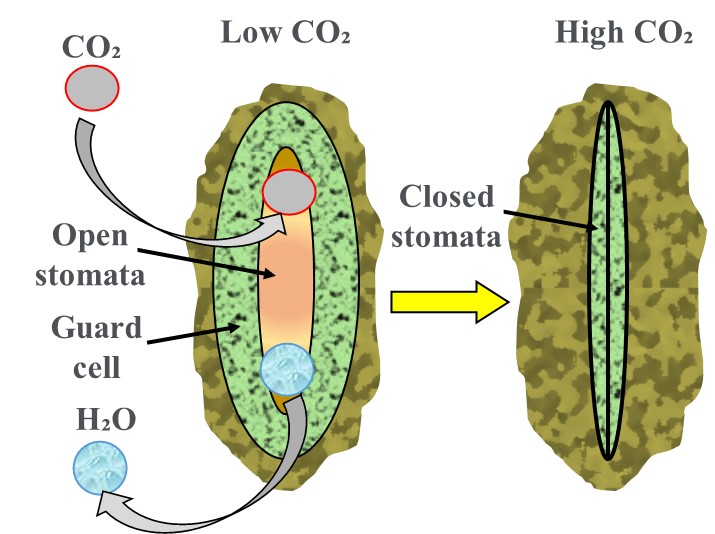


**Supplementary figure 10.** Schematic of a plant leaf stomatal activity under low and high CO_2_ availability.


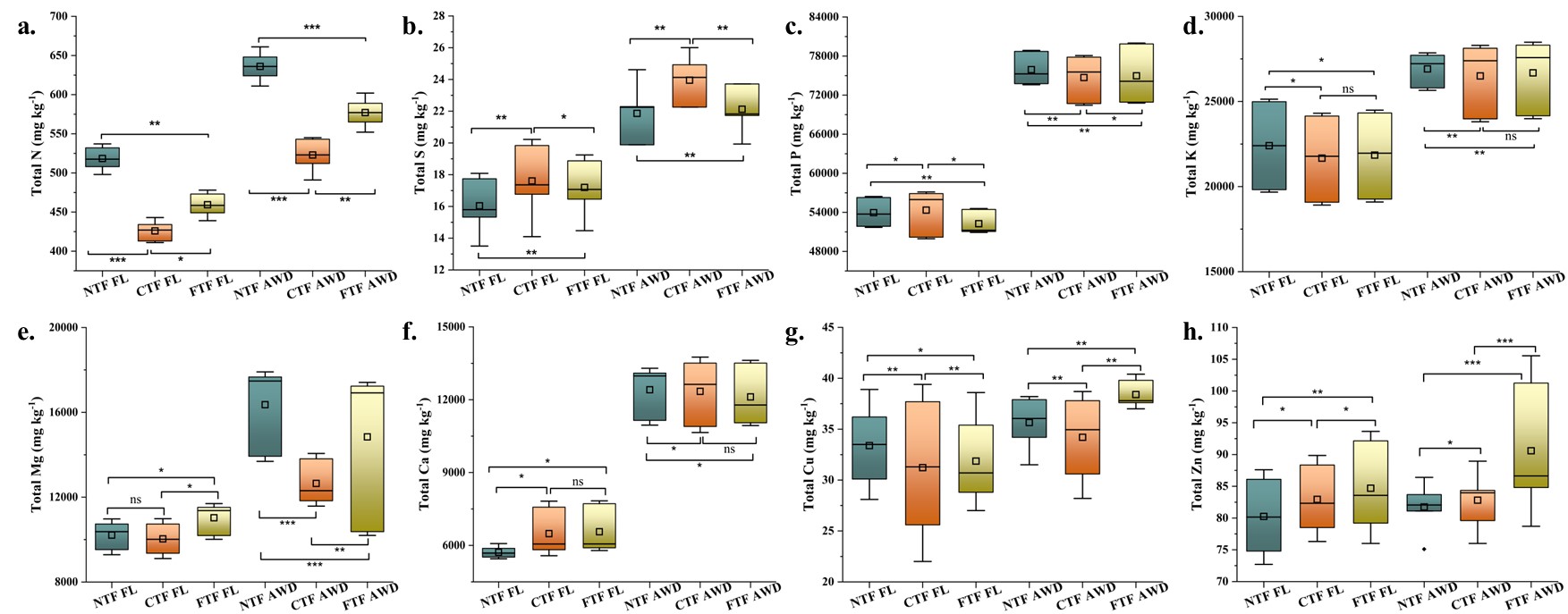


**Supplementary figure 11.** Total soil elemental concentrations analysed from three field setups with differential irrigation.


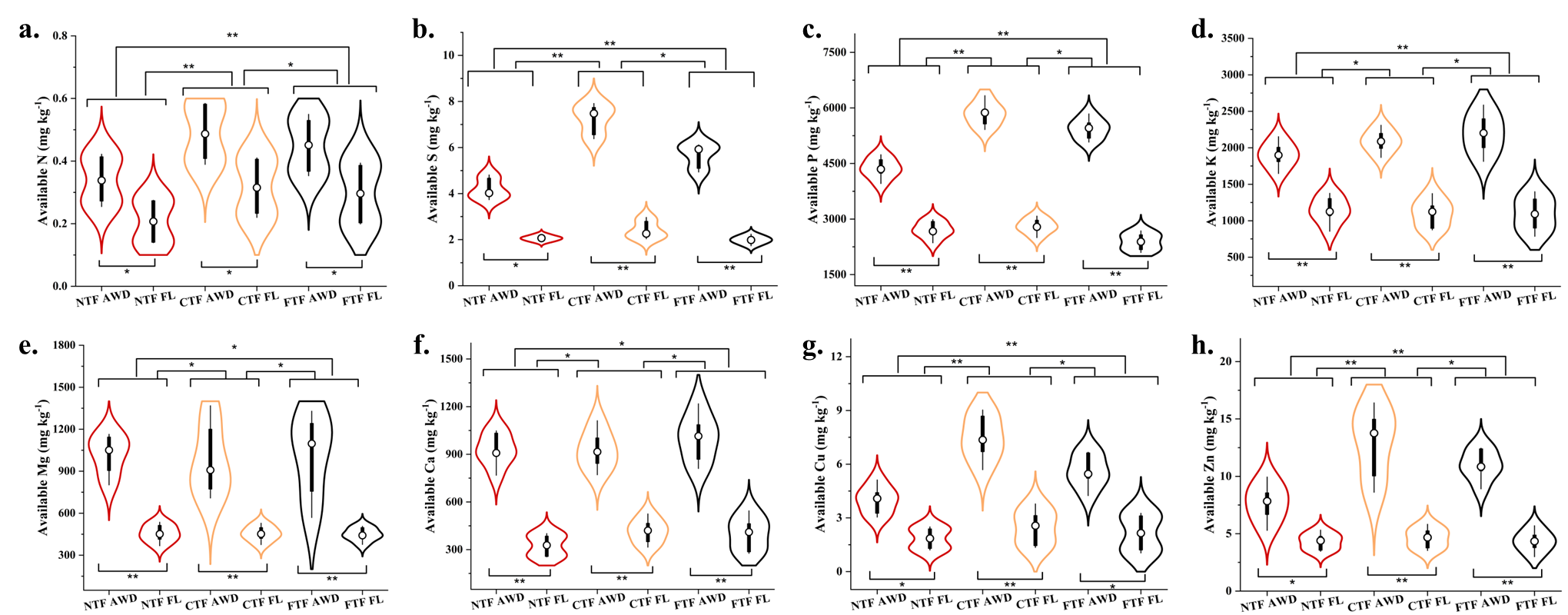


**Supplementary figure 12.** Bioavailable concentrations of selected soil elements N-S-P-K-Mg-Ca-Cu-Zn **(a-h)** from three field setups with varied tillage practices. Violin-box distribution plots are presented here. These are the average data of two-year soil sequential extraction analysis from 12 experimental sites. Distinct concentration differences and statistical significance variance have been found. ANOA with *p*<0.05 and *p*<0.01 significance levels are marked as ‘*’ and ‘**’, respectively, when compared within groups.


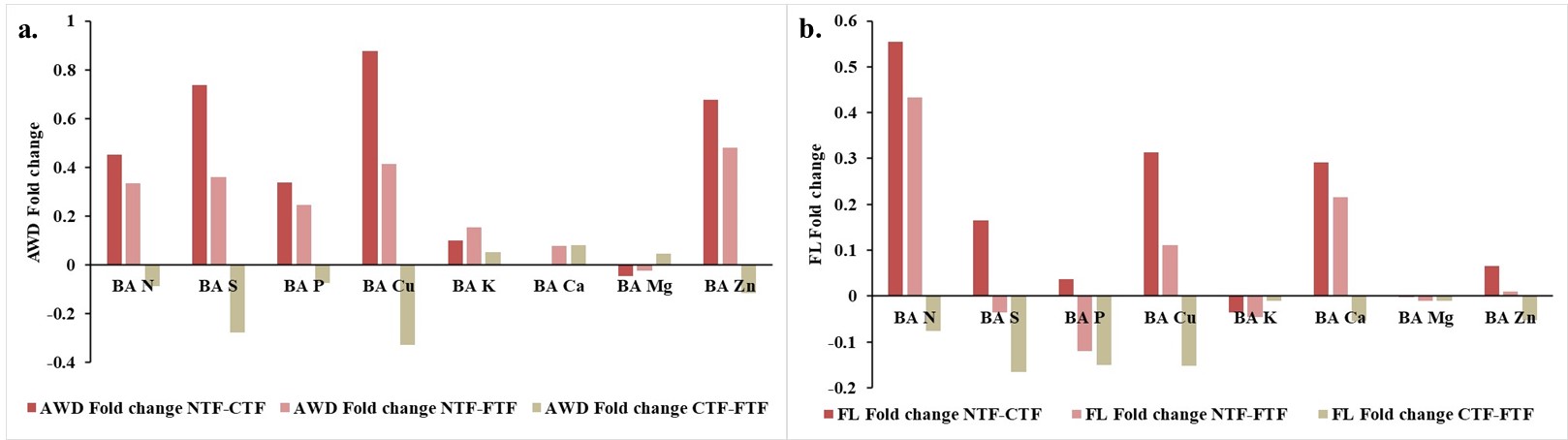


**Supplementary figure 13.** Fold change of the bioavailability in three field setups for the selected elements under AWD and FL irrigations.


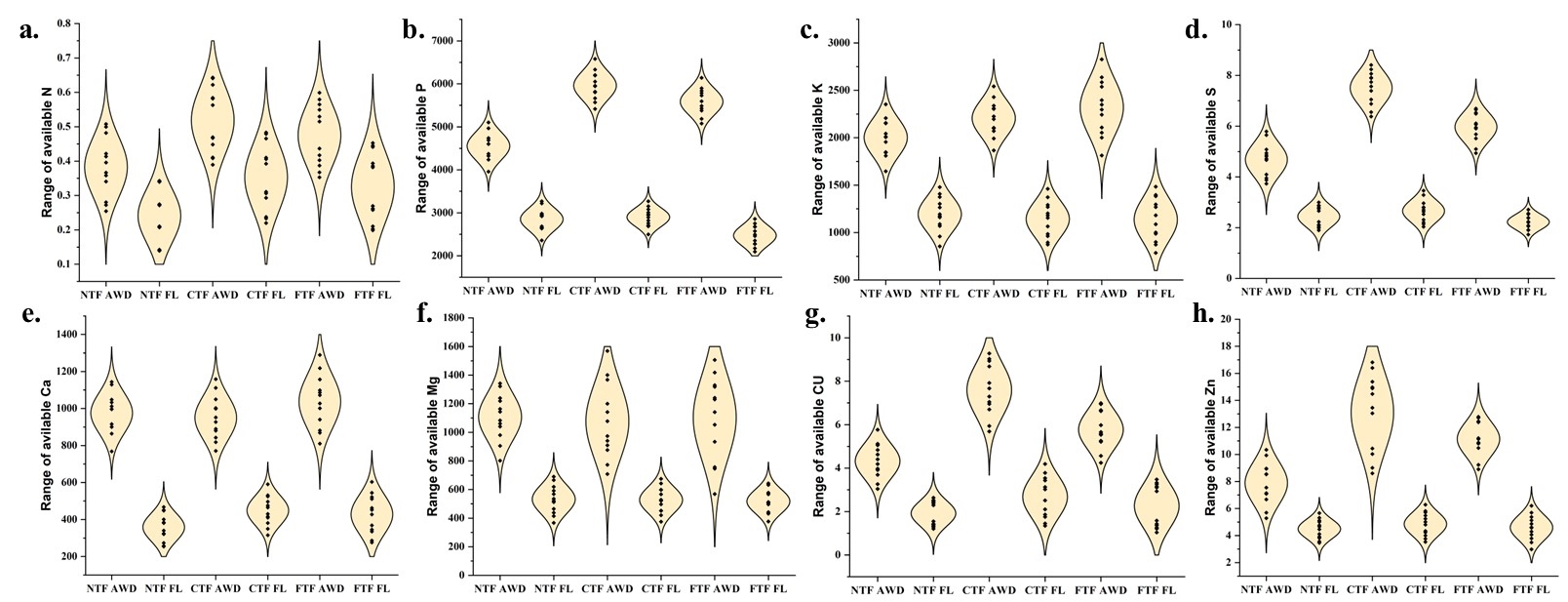


**Supplementary figure 14**. Average data trend of bioavailable elements in the normal distribution model.


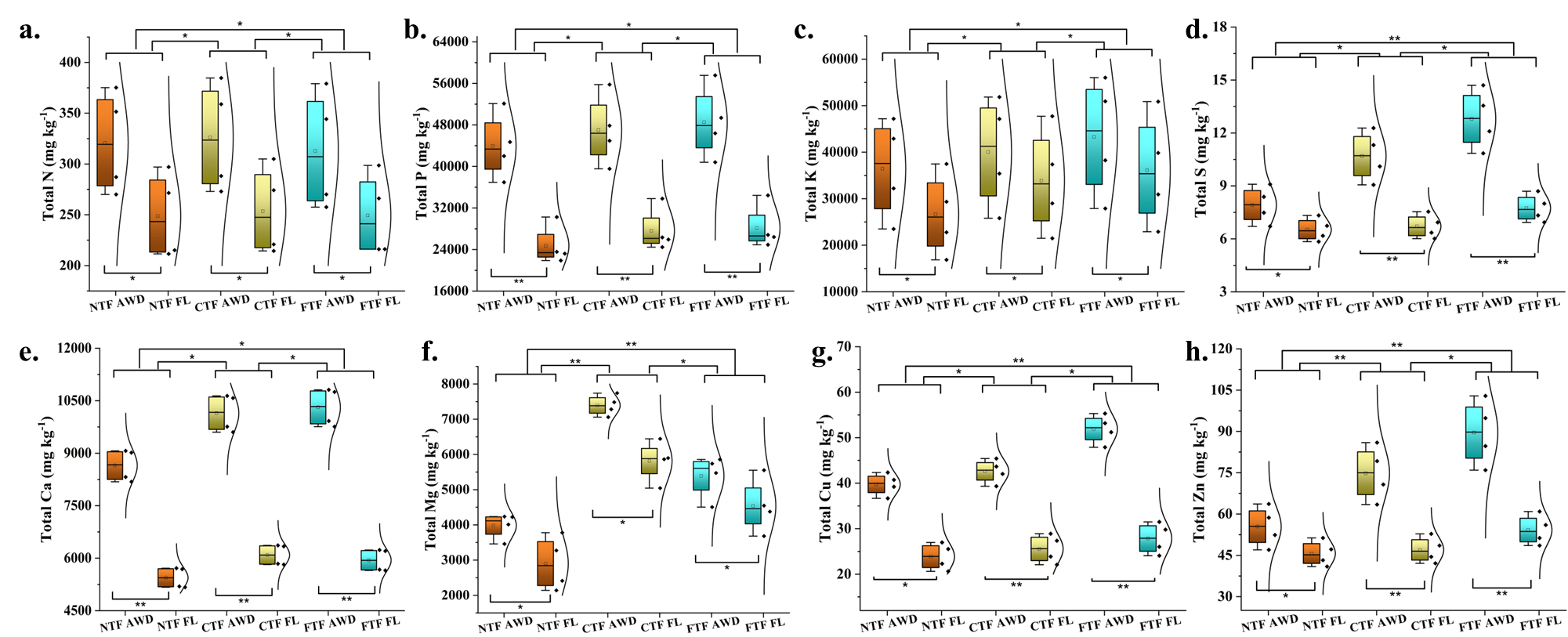


**Supplementary figure 15.** Total plant elemental concentrations analysed from three field setups with differential irrigation at the final harvest phase.


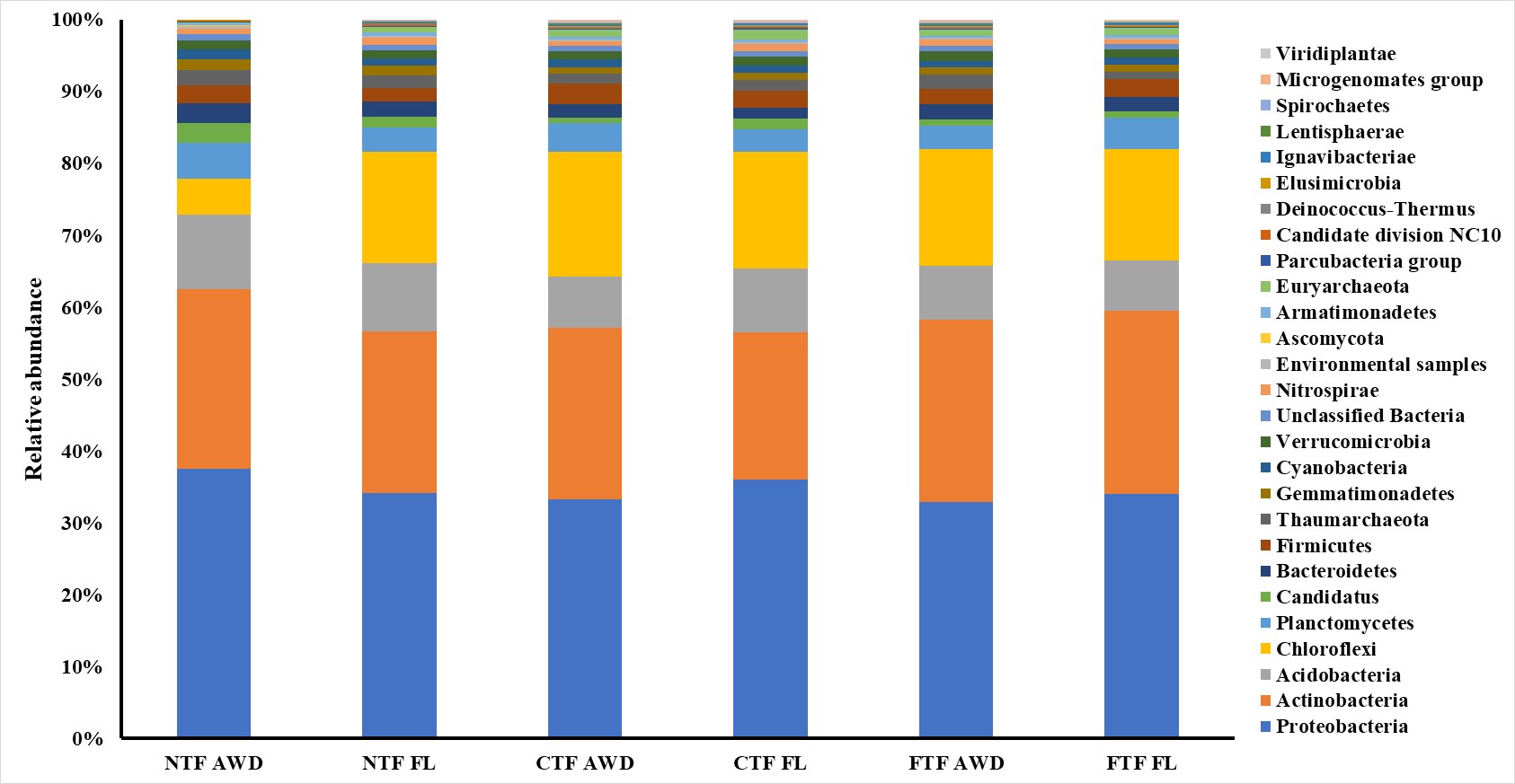


**Supplementary figure 16.** Microbial phyla relative abundance from the read-counts presented in stacked columns with a color-coded formation.


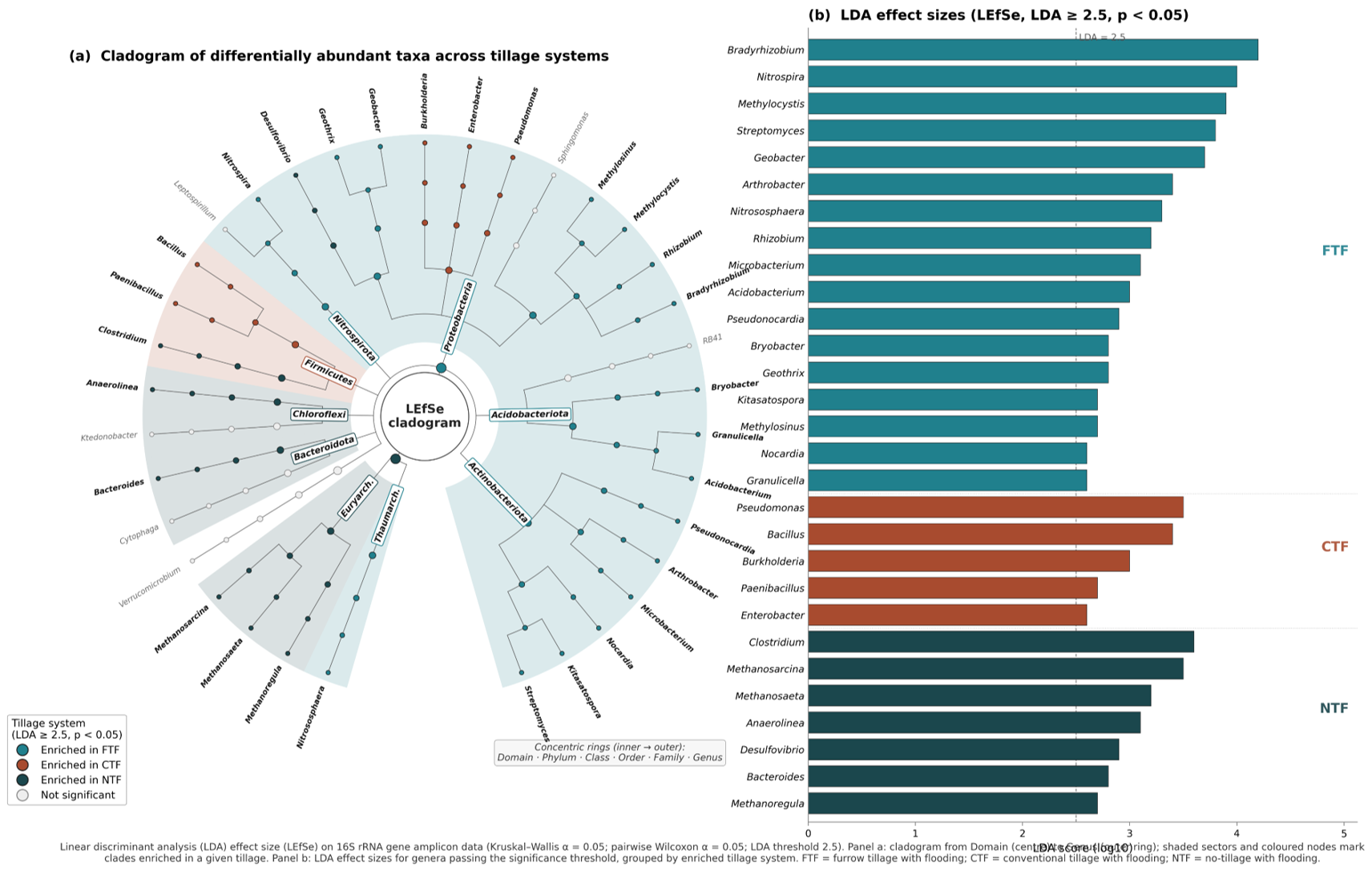


**Supplementary figure 17.** Microbial LEfSe cladogram structure under differential setups.


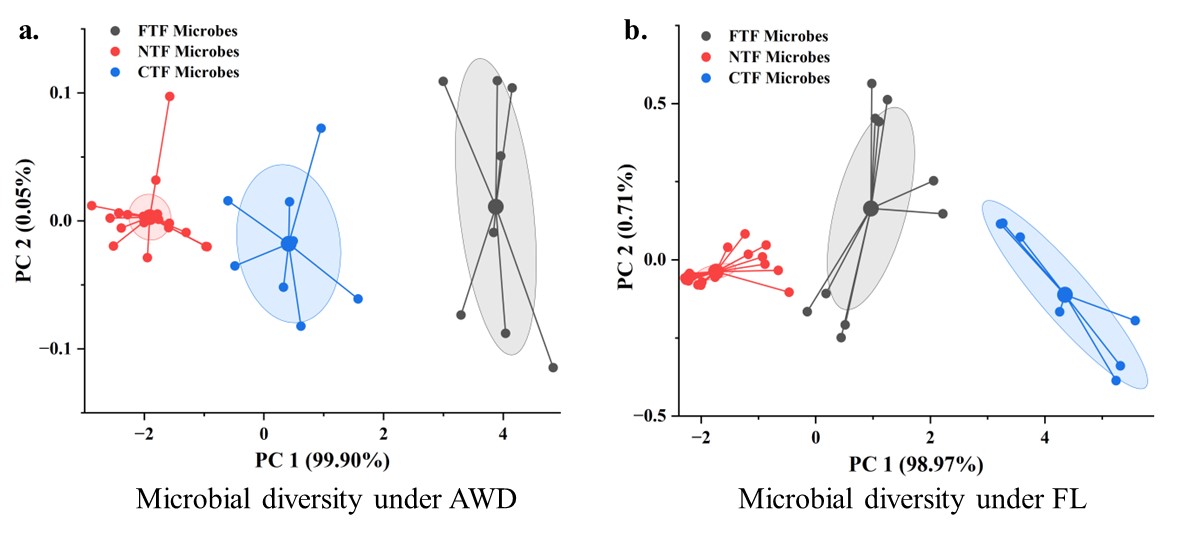


**Supplementary figure 18**. Machine-learning-based K-means cluster analysis for the statistical variance and significance analysis combined with principal component (PC) analysis.


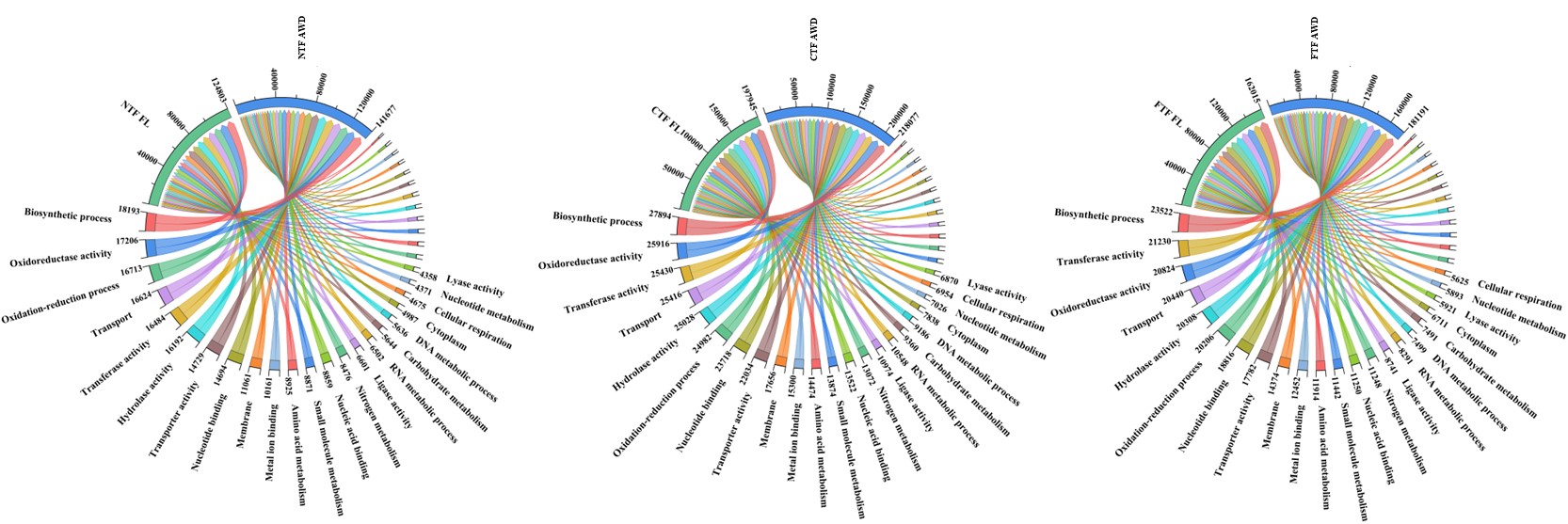


**Supplementary figure 19.** High frequency gene ontological terms found in microbial communities from the three field setups with varied irrigation.


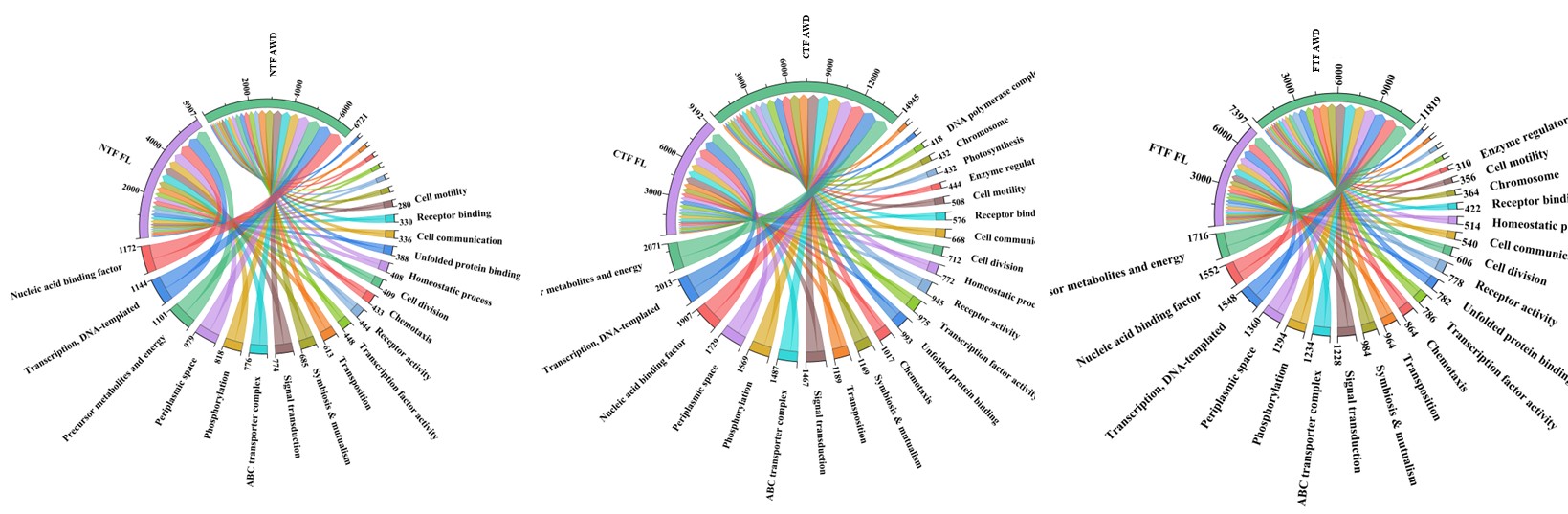


**Supplementary figure 20.** Low frequency gene ontological terms found in microbial communities from the three field setups with varied irrigation.


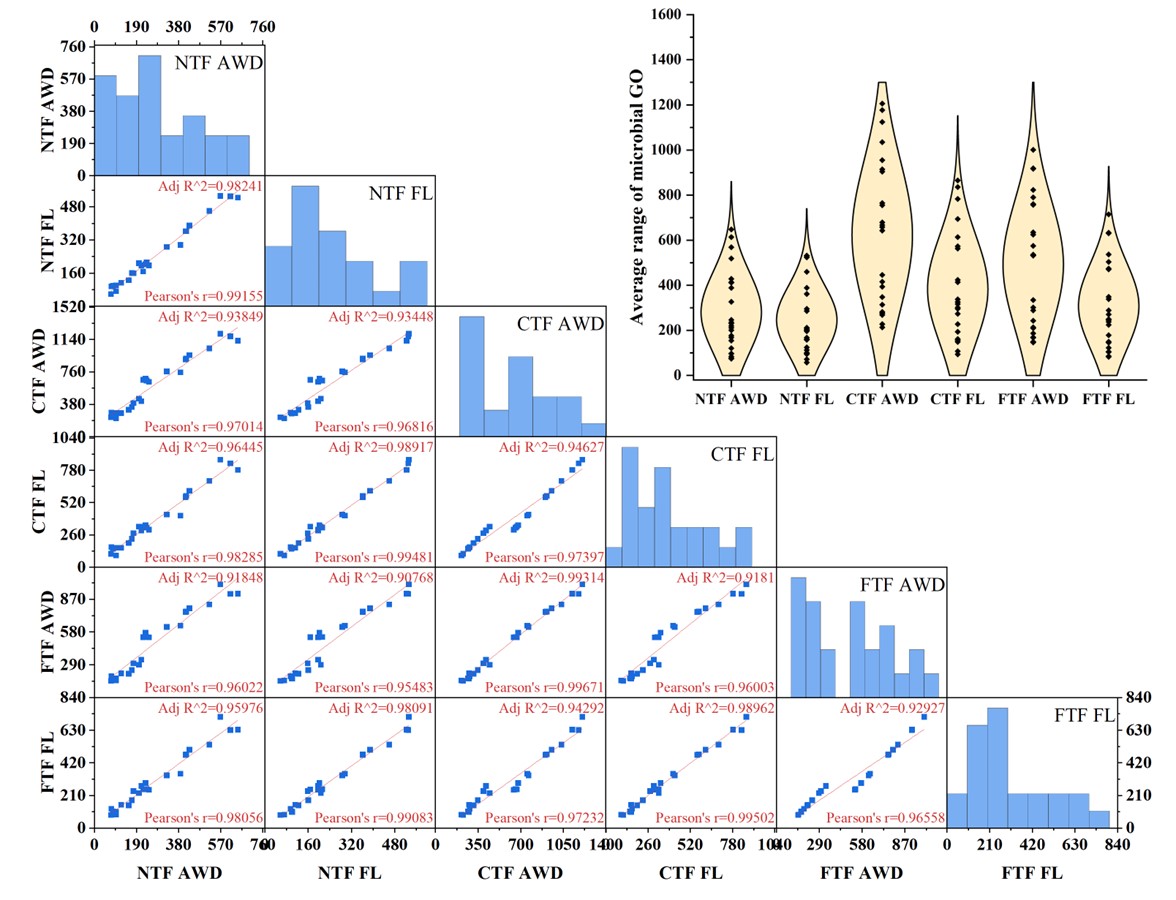


**Supplementary figure 21**. Statistical analysis of microbial GO distribution in differential setups with a multi-level scatter-matrix plot with linear fit. The violin plot shows the normal distribution mode of the average GO terms counted for these setups.


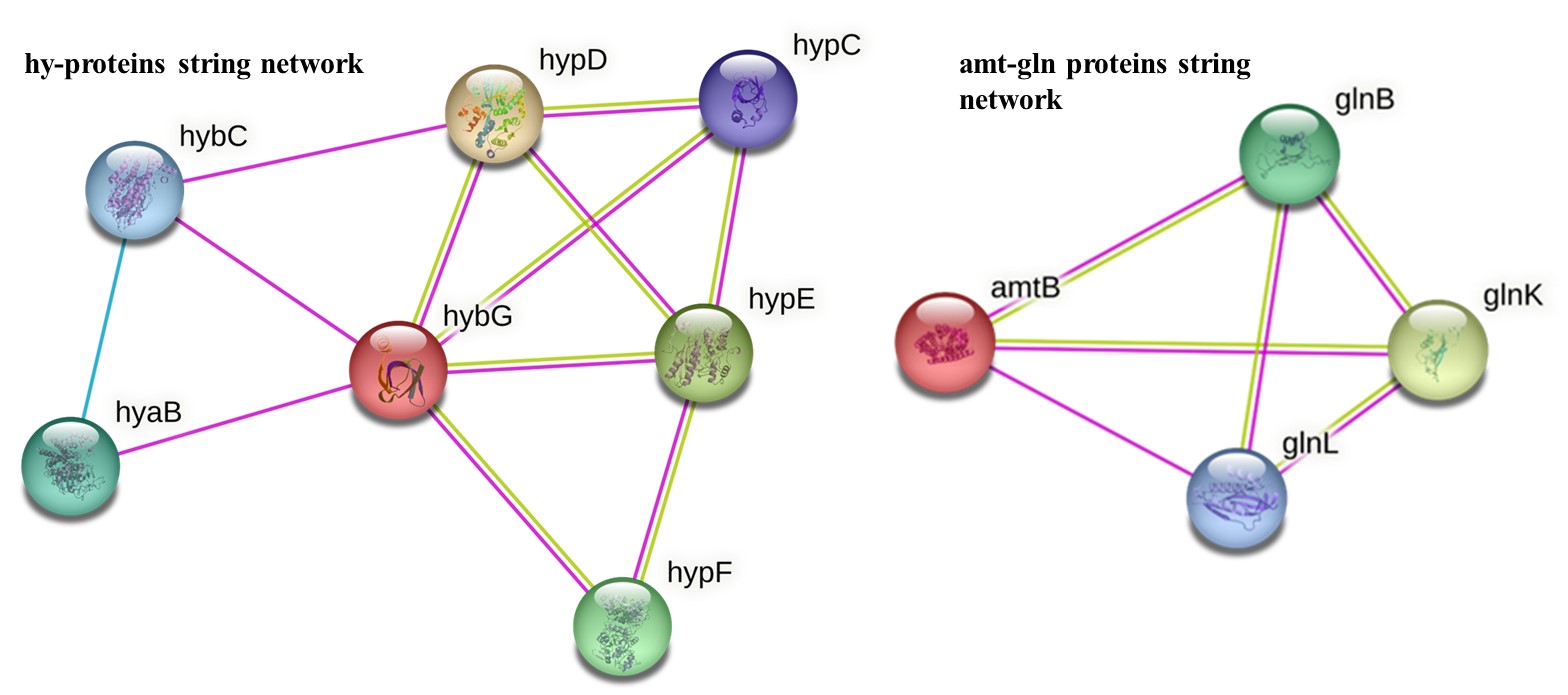


**Supplementary figure 22.** String networks of hyb-hyp proteins and amt-gln proteins that participate in different metal and gaseous molecule binding and transportation at the cellular level.


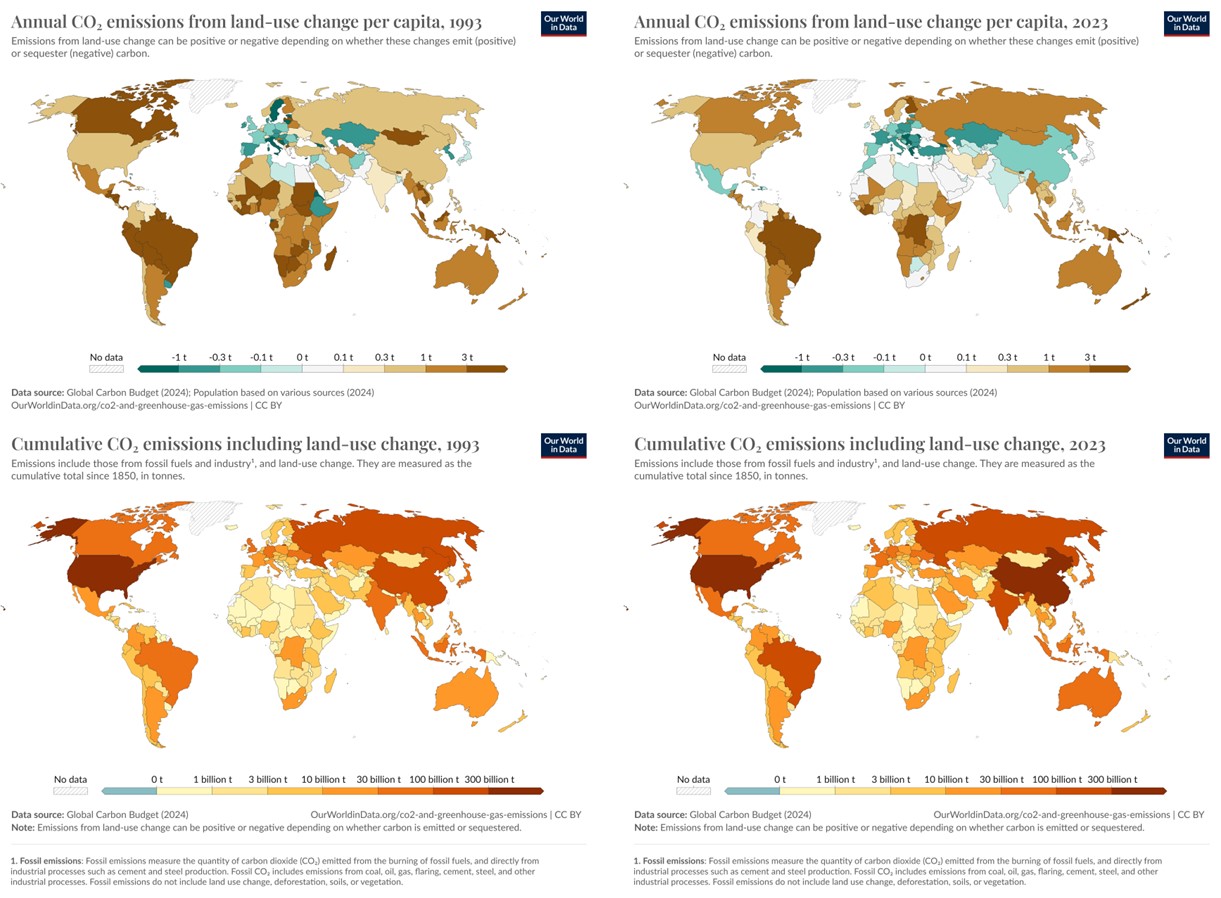


**Supplementary figure 23.** Annual and cumulative CO_2_ release global map within the span of 30 years (1993-2023).


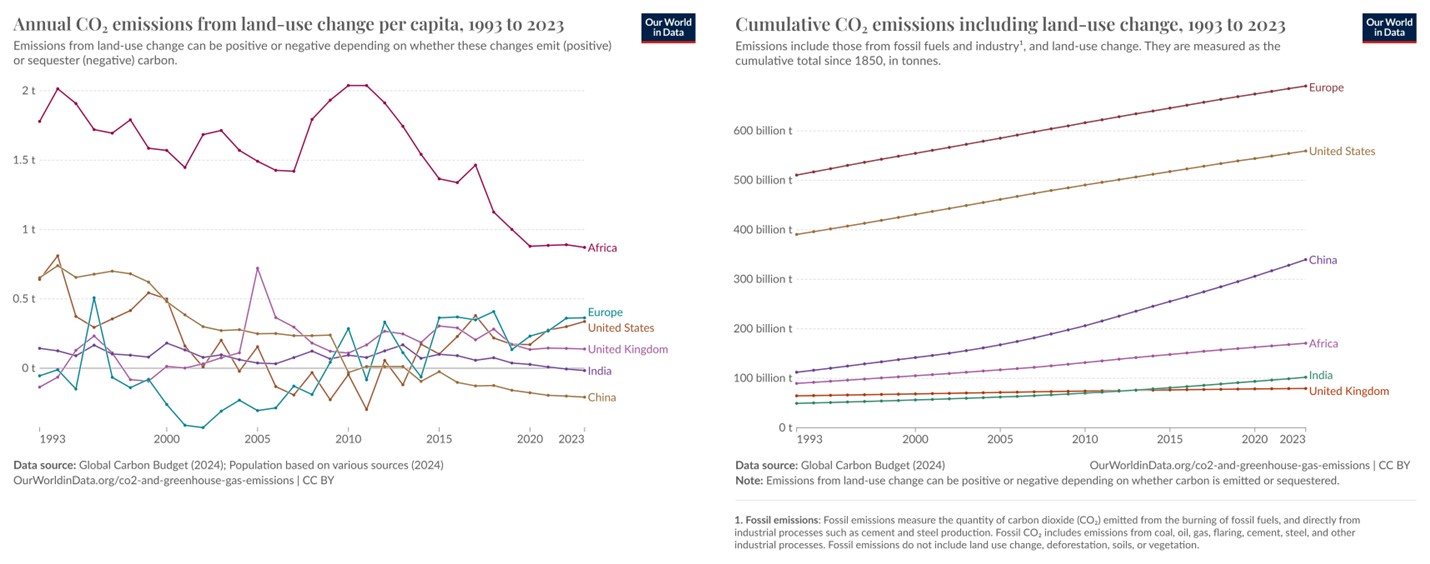


**Supplementary figure 24.** Annual and cumulative CO_2_ release trend chart within the span of 30 years (1993-2023).

| CO_2_ collection and field trial sites | | | | | | | | |
| --- | --- | --- | --- | --- | --- | --- | --- | --- |
|  |  |  | Coordinates |  |  |  |  | Coordinates |
|  |  |  | Latitude & Longitude | |  |  |  | Latitude & Longitude |
| 2022 | Area 1 | AWD 1st | 22.966965, 88.588591 | | 2023 | Area 1 | AWD 1st | 22.966965, 88.588591 |
|  |  | AWD 2nd | 22.965809, 88.588559 | |  |  | AWD 2nd | 22.965809, 88.588559 |
|  |  | AWD 3rd | 22.966194, 88.590125 | |  |  | AWD 3rd | 22.966194, 88.590125 |
|  |  | FL 1st | 22.966925, 88.587014 | |  |  | FL 1st | 22.966925, 88.587014 |
|  |  | Fl 2nd | 22.966422, 88.584933 | |  |  | Fl 2nd | 22.966422, 88.584933 |
|  |  | Fl 3rd | 22.965295, 88.586714 | |  |  | Fl 3rd | 22.965295, 88.586714 |
|  |  |  |  |  |  |  |  |  |
| 2022 | Area 2 | AWD 1st | 22.971983, 88.586649 | | 2023 | Area 2 | AWD 1st | 22.971983, 88.586649 |
|  |  | AWD 2nd | 22.971716, 88.588817 | |  |  | AWD 2nd | 22.971716, 88.588817 |
|  |  | AWD 3rd | 22.970155, 88.586317 | |  |  | AWD 3rd | 22.970155, 88.586317 |
|  |  | FL 1st | 22.974077, 88.582755 | |  |  | FL 1st | 22.974077, 88.582755 |
|  |  | Fl 2nd | 22.974255, 88.584321 | |  |  | Fl 2nd | 22.974255, 88.584321 |
|  |  | Fl 3rd | 22.972348, 88.584032 | |  |  | Fl 3rd | 22.972348, 88.584032 |
|  |  |  |  |  |  |  |  |  |
| 2022 | Area 3 | AWD 1st | 22.962342, 88.579354 | | 2023 | Area 3 | AWD 1st | 22.962342, 88.579354 |
|  |  | AWD 2nd | 22.962105, 88.581371 | |  |  | AWD 2nd | 22.962105, 88.581371 |
|  |  | AWD 3rd | 22.960563, 88.580169 | |  |  | AWD 3rd | 22.960563, 88.580169 |
|  |  | FL 1st | 22.962095, 88.576929 | |  |  | FL 1st | 22.962095, 88.576929 |
|  |  | Fl 2nd | 22.960771, 88.577680 | |  |  | Fl 2nd | 22.960771, 88.577680 |
|  |  | Fl 3rd | 22.960060, 88.579482 | |  |  | Fl 3rd | 22.960060, 88.579482 |
|  |  |  |  |  |  |  |  |  |
| 2022 | Area 4 | AWD 1st | 22.963092, 88.587357 | | 2023 | Area 4 | AWD 1st | 22.963092, 88.587357 |
|  |  | AWD 2nd | 22.963616, 88.589160 | |  |  | AWD 2nd | 22.963616, 88.589160 |
|  |  | AWD 3rd | 22.961848, 88.588634 | |  |  | AWD 3rd | 22.961848, 88.588634 |
|  |  | FL 1st | 22.960544, 88.585308 | |  |  | FL 1st | 22.960544, 88.585308 |
|  |  | Fl 2nd | 22.960988, 88.588430 | |  |  | Fl 2nd | 22.960988, 88.588430 |
|  |  | Fl 3rd | 22.959329, 88.586692 | |  |  | Fl 3rd | 22.959329, 88.586692 |
|  |  |  |  |  |  |  |  |  |
| 2022 | Area 5 | AWD 1st | 22.952700, 88.585534 | | 2023 | Area 5 | AWD 1st | 22.952700, 88.585534 |
|  |  | AWD 2nd | 22.953757, 88.587797 | |  |  | AWD 2nd | 22.953757, 88.587797 |
|  |  | AWD 3rd | 22.952502, 88.586907 | |  |  | AWD 3rd | 22.952502, 88.586907 |
|  |  | FL 1st | 22.953895, 88.580781 | |  |  | FL 1st | 22.953895, 88.580781 |
|  |  | Fl 2nd | 22.953885, 88.583109 | |  |  | Fl 2nd | 22.953885, 88.583109 |
|  |  | Fl 3rd | 22.952650, 88.581371 | |  |  | Fl 3rd | 22.952650, 88.581371 |
|  |  |  |  |  |  |  |  |  |
| 2022 | Area 6 | AWD 1st | 22.966984, 88.602335 | | 2023 | Area 6 | AWD 1st | 22.966984, 88.602335 |
|  |  | AWD 2nd | 22.967024, 88.604384 | |  |  | AWD 2nd | 22.967024, 88.604384 |
|  |  | AWD 3rd | 22.965858, 88.604363 | |  |  | AWD 3rd | 22.965858, 88.604363 |
|  |  | FL 1st | 22.966154, 88.599513 | |  |  | FL 1st | 22.966154, 88.599513 |
|  |  | Fl 2nd | 22.965957, 88.601445 | |  |  | Fl 2nd | 22.965957, 88.601445 |
|  |  | Fl 3rd | 22.964742, 88.600243 | |  |  | Fl 3rd | 22.964742, 88.600243 |
|  |  |  |  |  |  |  |  |  |
| 2022 | Area 7 | AWD 1st | 22.983115, 88.578635 | | 2023 | Area 7 | AWD 1st | 22.983115, 88.578635 |
|  |  | AWD 2nd | 22.983085, 88.580159 | |  |  | AWD 2nd | 22.983085, 88.580159 |
|  |  | AWD 3rd | 22.981337, 88.579601 | |  |  | AWD 3rd | 22.981337, 88.579601 |
|  |  | FL 1st | 22.983757, 88.581929 | |  |  | FL 1st | 22.983757, 88.581929 |
|  |  | Fl 2nd | 22.983549, 88.584128 | |  |  | Fl 2nd | 22.983549, 88.584128 |
|  |  | Fl 3rd | 22.981919, 88.583131 | |  |  | Fl 3rd | 22.981919, 88.583131 |
|  |  |  |  |  |  |  |  |  |
| 2022 | Area 8 | AWD 1st | 22.988912, 88.613879 | | 2023 | Area 8 | AWD 1st | 22.988912, 88.613879 |
|  |  | AWD 2nd | 22.988586, 88.616594 | |  |  | AWD 2nd | 22.988586, 88.616594 |
|  |  | AWD 3rd | 22.987520, 88.615113 | |  |  | AWD 3rd | 22.987520, 88.615113 |
|  |  | FL 1st | 22.986206, 88.612828 | |  |  | FL 1st | 22.986206, 88.612828 |
|  |  | Fl 2nd | 22.985673, 88.615811 | |  |  | Fl 2nd | 22.985673, 88.615811 |
|  |  | Fl 3rd | 22.984359, 88.616411 | |  |  | Fl 3rd | 22.984359, 88.616411 |
|  |  |  |  |  |  |  |  |  |
| 2022 | Area 9 | AWD 1st | 22.994058, 88.614673 | | 2023 | Area 9 | AWD 1st | 22.994058, 88.614673 |
|  |  | AWD 2nd | 22.994364, 88.617034 | |  |  | AWD 2nd | 22.994364, 88.617034 |
|  |  | AWD 3rd | 22.992656, 88.616519 | |  |  | AWD 3rd | 22.992656, 88.616519 |
|  |  | FL 1st | 22.994572, 88.619673 | |  |  | FL 1st | 22.994572, 88.619673 |
|  |  | Fl 2nd | 22.994483, 88.621744 | |  |  | Fl 2nd | 22.994483, 88.621744 |
|  |  | Fl 3rd | 22.993456, 88.620113 | |  |  | Fl 3rd | 22.993456, 88.620113 |
|  |  |  |  |  |  |  |  |  |
| 2022 | Area 10 | AWD 1st | 22.970590, 88.603998 | | 2023 | Area 10 | AWD 1st | 22.970590, 88.603998 |
|  |  | AWD 2nd | 22.970135, 88.606004 | |  |  | AWD 2nd | 22.970135, 88.606004 |
|  |  | AWD 3rd | 22.968683, 88.604792 | |  |  | AWD 3rd | 22.968683, 88.604792 |
|  |  | FL 1st | 22.970777, 88.607796 | |  |  | FL 1st | 22.970777, 88.607796 |
|  |  | Fl 2nd | 22.970234, 88.610060 | |  |  | Fl 2nd | 22.970234, 88.610060 |
|  |  | Fl 3rd | 22.969009, 88.608311 | |  |  | Fl 3rd | 22.969009, 88.608311 |
|  |  |  |  |  |  |  |  |  |
| 2022 | Area 11 | AWD 1st | 22.952179, 88.569017 | | 2023 | Area 11 | AWD 1st | 22.952179, 88.569017 |
|  |  | AWD 2nd | 22.952742, 88.571431 | |  |  | AWD 2nd | 22.952742, 88.571431 |
|  |  | AWD 3rd | 22.951033, 88.570744 | |  |  | AWD 3rd | 22.951033, 88.570744 |
|  |  | FL 1st | 22.951428, 88.566527 | |  |  | FL 1st | 22.951428, 88.566527 |
|  |  | Fl 2nd | 22.951340, 88.564864 | |  |  | Fl 2nd | 22.951340, 88.564864 |
|  |  | Fl 3rd | 22.949917, 88.566045 | |  |  | Fl 3rd | 22.949917, 88.566045 |
|  |  |  |  |  |  |  |  |  |
| 2022 | Area 12 | AWD 1st | 22.955706, 88.582996 | | 2023 | Area 12 | AWD 1st | 22.955706, 88.582996 |
|  |  | AWD 2nd | 22.955242, 88.585260 | |  |  | AWD 2nd | 22.955242, 88.585260 |
|  |  | AWD 3rd | 22.953632, 88.582664 | |  |  | AWD 3rd | 22.953632, 88.582664 |
|  |  | FL 1st | 22.955183, 88.578705 | |  |  | FL 1st | 22.955183, 88.578705 |
|  |  | Fl 2nd | 22.955173, 88.580314 | |  |  | Fl 2nd | 22.955173, 88.580314 |
|  |  | Fl 3rd | 22.953661, 88.580003 | |  |  | Fl 3rd | 22.953661, 88.580003 |

**Supplementary table 1**. Coordinates of the selected 12 experimental sites where field trials were conducted in two consecutive years. Two irrigation regimes, alternate wetting and drying (AWD) and flooded (FL), were combined with three tillage practices.

| Soil CO_2_ | | |
| --- | --- | --- |
| Mann-Whitney U Test at *P*<0.05 | | |
|  | U values | Exact *P* values |
| NTF AWD to CTF AWD | 19 | 0.00382 |
| NTF FL to CTF FL | 30 | 0.00414 |
| NTF AWD to FTF AWD | 77 | 0.0069 |
| NTF FL to FTF FL | 72 | 0.00732 |
| CTF AWD to FTF AWD | 32 | 0.08242 |
| CTF FL to FTF FL | 41 | 0.07928 |

**Supplementary table 2**. Post-hoc analysis of soil CO_2_ release from the fields with different combinations.

| Soil elements | | |
| --- | --- | --- |
| Mann-Whitney U Test at *P*<0.05 | | |
|  | U values | Exact *P* values |
| NTF AWD to CTF AWD | 29 | 0.00472 |
| NTF FL to CTF FL | 34 | 0.00428 |
| NTF AWD to FTF AWD | 81 | 0.00688 |
| NTF FL to FTF FL | 56 | 0.00722 |
| CTF AWD to FTF AWD | 52 | 0.00826 |
| CTF FL to FTF FL | 61 | 0.00794 |

| Response | Tillage main | Irrigation main | Tillage × Irrigation |
| --- | --- | --- | --- |
| Total soil C | F₂,₂₉ = 546.32, p < 0.0001 | F₁,₂₉ = 79.23, p < 0.0001 | **F₂,₂₉ = 20.19, p < 0.0001** |
| Labile C | F₂,₂₉ = 1 116.52, p < 0.0001 | F₁,₂₉ = 153.22, p < 0.0001 | **F₂,₂₉ = 5.70, p = 0.0081** |
| CO₂ flux | F₂,₂₉ = 4 701.74, p < 0.0001 | F₁,₂₉ = 139.73, p < 0.0001 | **F₂,₂₉ = 20.61, p < 0.0001** |

**Supplementary table 3**. Post-hoc analysis of soil elemental bioavailability from the fields with different combinations.

| Soil Microbes | | |
| --- | --- | --- |
| Mann-Whitney U Test at *P*<0.05 | | |
|  | U values | Exact *P* values |
| NTF AWD to CTF AWD | 22 | 0.00212 |
| NTF FL to CTF FL | 30 | 0.00218 |
| NTF AWD to FTF AWD | 71 | 0.00422 |
| NTF FL to FTF FL | 42 | 0.00436 |
| CTF AWD to FTF AWD | 49 | 0.00596 |
| CTF FL to FTF FL | 53 | 0.00608 |

**Supplementary table 4**. Post-hoc analysis of soil microbial diversity changes in the fields with different combinations.

| Years | Tillage practice | Variogram model | Coefficient of determination (%) | Lag distance (m) | Maximum distance (m) |
| --- | --- | --- | --- | --- | --- |
| 2022 | NTF | -0.0231004 + 0.00699175 ln(1 + x) | 53.90 % | 171.50 | 3822.68 |
|  | CTF | -0.171484 + 0.0439949 ln(1 + x) | 66.33 % | 171.50 | 3822.68 |
|  | FTF | -0.0911852 + 0.0186986 ln(1 + x) | 81.74% | 171.50 | 3822.68 |
| 2023 | NTF | -0.0466559 + 0.0151919 ln(1 + x) | 35.54% | 171.50 | 3822.68 |
|  | CTF | -0.288862 + 0.0823526 ln(1 + x) | 56.32% | 171.50 | 3822.68 |
|  | FTF | -0.152707 + 0.0363219 ln(1 + x) | 58.41% | 171.50 | 3822.68 |

**Supplementary table 5**. Variogram models for Ordinary Kriging corresponding to various tillage practices.

| Gene | Forward Primer (5'-3') | | | Reverse Primer (5'-3') | Amplicon | Efficiency |
| --- | --- | --- | --- | --- | --- | --- |
| OsRbcS2 | GCAAGTGTTGGAGACACCCT | | | GGTCCATGTCACCACCAAAG | 120 bp | 95% |
| OsRbcS3 | GCAAGTGTTGGAGACACCGT | | | GGTCCATGTCGCCACCAAAG | 115 bp | 92% |
| OsRbcS4 | GCAAGTGTCGGAGACACCCT | | | GGTCCATGTCACCGCCAAAG | 118 bp | 94% |
| OsRbcS5 | GCAAGTGTTGGAGACACCAT | | | GGTCCATGTCACCACCGATG | 122 bp | 96% |
| RbcL-1 | ATGTCACCACAAACAGAGACTAAAGC | | | TCACAAGCTCCCCAATTCTTTAC | 180 bp | 90% |
| RbcL-2 | ATGTCACCACAAACAGAAACTAAAGC | | | TCACAAGCTCCCCAATTCTGTAC | 185 bp | 88% |
| FBPase-1 | TCCATGGCTGATGAGATGGT | | | CGAAGCTCTTGGCAATGAAG | 150 bp | 93% |
| FBPase-2 | TCCATGGCTGATGAGATCGT | | | CGAAGCTCTTGGCGATGAAG | 155 bp | 91% |
| GOX2-1 | GAGGCTTTGGATGACCTGAA | | | TGGCTCTGGATGGGTACTTG | 140 bp | 89% |
| GOX2-2 | GAGGCTTTGGATGACTTGAA | | | TGGCTCTGGATGGGTGCTTG | 145 bp | 87% |
| GGT2-1 | ATCGACGTCAACGAGTTCGT | | | GCGTCCTCGTAGTTGGTGAA | 160 bp | 92% |
| GGT2-2 | ATCGACGTCAACGAGATCGT | | | GCGTCCTCGTAGATGGTGAA | 165 bp | 94% |
| GLDP1-1 | CGACATCAAGGACTACGTGG | | | GAACTTCCTCGAGGGTGTCC | 170 bp | 95% |
| GLDP1-2 | CGACATCAAGGACTACATGG | | | GAACTTCCTCGAGGATGTCC | 175 bp | 93% |
| Actin | | CTTGCACCAAGCAGCATGAA | CCGATCCAGACACTGTACTTCCTT | | 150 bp |  |

**Supplementary table 6**. Primer pairs for the QPCR analysis of selected genes. Oryza sativa (Os) RbcS (Ribulose bisphosphate carboxylase small subunit), RbcL (Ribulose bisphosphate carboxylase large subunit), FBPase (Fructose-1,6-bisphosphatase), GOX2 (Glycolate oxidase 2), GGT2 (γ-glutamyltranspeptidase 2), GLDP1 (Glycine decarboxylase P-protein 1) were targeted while Actin was selected as the reference gene.

| Root dry weight (g) | | | | | | | | | |
| --- | --- | --- | --- | --- | --- | --- | --- | --- | --- |
|  |  | AWD 2022 | SD | AWD 2023 | SD | FL 2022 | SD | FL 2023 | SD |
| NTF | R1 | 3.81 | 0.14 | 4.3 | 0.2 | 3.75 | 0.23 | 3.79 | 0.21 |
|  | R2 | 4.02 | 0.21 | 4.26 | 0.17 | 3.54 | 0.19 | 4.02 | 0.15 |
|  | R3 | 4.14 | 0.17 | 4.34 | 0.15 | 3.67 | 0.14 | 3.84 | 0.17 |
| CTF | R1 | 5.77 | 0.16 | 5.55 | 0.19 | 4.88 | 0.21 | 4.81 | 0.13 |
|  | R2 | 5.47 | 0.18 | 5.39 | 0.14 | 4.67 | 0.16 | 5.24 | 0.12 |
|  | R3 | 5.13 | 0.17 | 5.76 | 0.13 | 4.95 | 0.17 | 5.27 | 0.18 |
| FTF | R1 | 4.92 | 0.22 | 5.4 | 0.18 | 4.84 | 0.15 | 4.88 | 0.16 |
|  | R2 | 5.57 | 0.18 | 4.75 | 0.14 | 4.72 | 0.19 | 4.81 | 0.15 |
|  | R3 | 5.2 | 0.13 | 5.31 | 0.15 | 4.89 | 0.14 | 4.73 | 0.19 |
|  | Shoot dry weight (g) | | | | | | | | |
|  |  | AWD 2022 | SD | AWD 2023 | SD | FL 2022 | SD | FL 2023 | SD |
| NTF | R1 | 26.19 | 0.43 | 23.31 | 0.49 | 23.03 | 0.38 | 21.65 | 0.47 |
|  | R2 | 25.00 | 0.65 | 26.42 | 0.73 | 23.01 | 0.58 | 20.24 | 0.70 |
|  | R3 | 22.75 | 0.53 | 25.21 | 0.59 | 21.22 | 0.47 | 21.06 | 0.57 |
| CTF | R1 | 31.11 | 0.50 | 30.79 | 0.56 | 26.41 | 0.44 | 28.70 | 0.53 |
|  | R2 | 31.02 | 0.56 | 29.69 | 0.62 | 30.03 | 0.49 | 30.62 | 0.60 |
|  | R3 | 29.67 | 0.53 | 31.05 | 0.59 | 31.52 | 0.47 | 28.67 | 0.57 |
| FTF | R1 | 30.00 | 0.68 | 28.43 | 0.76 | 25.02 | 0.60 | 25.86 | 0.73 |
|  | R2 | 31.63 | 0.56 | 30.45 | 0.62 | 27.51 | 0.49 | 24.69 | 0.60 |
|  | R3 | 30.65 | 0.40 | 29.81 | 0.45 | 25.80 | 0.36 | 26.83 | 0.43 |
|  | Root length (cm) | | | | | | | | |
|  |  | AWD 2022 | SD | AWD 2023 | SD | FL 2022 | SD | FL 2023 | SD |
| NTF | R1 | 18.90 | 0.32 | 19.10 | 0.39 | 17.50 | 0.38 | 17.70 | 0.29 |
|  | R2 | 20.00 | 0.48 | 19.70 | 0.58 | 18.20 | 0.57 | 17.80 | 0.44 |
|  | R3 | 19.40 | 0.39 | 18.90 | 0.47 | 18.20 | 0.46 | 19.30 | 0.35 |
| CTF | R1 | 26.40 | 0.37 | 24.60 | 0.44 | 22.90 | 0.43 | 24.70 | 0.33 |
|  | R2 | 24.50 | 0.41 | 24.00 | 0.50 | 22.50 | 0.48 | 22.70 | 0.37 |
|  | R3 | 25.30 | 0.39 | 24.20 | 0.47 | 23.30 | 0.46 | 23.50 | 0.35 |
| FTF | R1 | 22.60 | 0.50 | 23.10 | 0.61 | 20.20 | 0.59 | 21.40 | 0.46 |
|  | R2 | 23.80 | 0.41 | 24.10 | 0.50 | 21.60 | 0.48 | 20.20 | 0.37 |
|  | R3 | 21.60 | 0.30 | 22.90 | 0.36 | 21.30 | 0.35 | 23.00 | 0.27 |
|  | Shoot length (cm) | | | | | | | | |
|  |  | AWD 2022 | SD | AWD 2023 | SD | FL 2022 | SD | FL 2023 | SD |
| NTF | R1 | 86.00 | 2.69 | 88.00 | 3.61 | 78.80 | 2.80 | 80.30 | 2.69 |
|  | R2 | 84.60 | 4.04 | 90.00 | 5.41 | 82.10 | 4.20 | 85.80 | 4.03 |
|  | R3 | 82.10 | 3.27 | 89.00 | 4.38 | 81.20 | 3.40 | 80.70 | 3.26 |
| CTF | R1 | 100.90 | 3.08 | 95.60 | 4.12 | 106.10 | 3.20 | 99.90 | 3.07 |
|  | R2 | 104.70 | 3.46 | 105.10 | 4.64 | 95.60 | 3.60 | 94.20 | 3.45 |
|  | R3 | 104.10 | 3.27 | 109.00 | 4.38 | 102.80 | 3.40 | 104.20 | 3.26 |
| FTF | R1 | 93.30 | 4.23 | 105.80 | 5.67 | 96.20 | 4.40 | 95.20 | 4.22 |
|  | R2 | 101.30 | 3.46 | 95.20 | 4.64 | 101.80 | 3.60 | 94.10 | 3.45 |
|  | R3 | 99.10 | 2.50 | 102.00 | 3.35 | 94.30 | 2.60 | 92.50 | 2.50 |
| Tiller numbers | | | | | | | | | |
|  |  | AWD 2022 | SD | AWD 2023 | SD | FL 2022 | SD | FL 2023 | SD |
| NTF | R1 | 11.00 | 0.16 | 113.00 | 0.17 | 10.00 | 0.15 | 11.00 | 0.16 |
|  | R2 | 12.00 | 0.24 | 12.00 | 0.26 | 11.00 | 0.22 | 10.00 | 0.24 |
|  | R3 | 10.00 | 0.19 | 11.00 | 0.21 | 10.00 | 0.18 | 12.00 | 0.19 |
| CTF | R1 | 16.00 | 0.18 | 17.00 | 0.20 | 13.00 | 0.17 | 14.00 | 0.18 |
|  | R2 | 17.00 | 0.21 | 16.00 | 0.22 | 15.00 | 0.19 | 15.00 | 0.20 |
|  | R3 | 16.00 | 0.19 | 16.00 | 0.21 | 14.00 | 0.18 | 13.00 | 0.19 |
| FTF | R1 | 14.00 | 0.25 | 15.00 | 0.27 | 14.00 | 0.24 | 13.00 | 0.25 |
|  | R2 | 14.00 | 0.21 | 14.00 | 0.22 | 13.00 | 0.19 | 14.00 | 0.20 |
|  | R3 | 16.00 | 0.15 | 15.00 | 0.16 | 13.00 |  | 12.00 | 0.15 |
|  | Panicle numbers | | | | | | | | |
|  |  | AWD 2022 | SD | AWD 2023 | SD | FL 2022 | SD | FL 2023 | SD |
| NTF | R1 | 9.00 | 0.13 | 10.00 | 0.16 | 8.00 | 0.14 | 9.00 | 0.13 |
|  | R2 | 10.00 | 0.19 | 10.00 | 0.24 | 7.00 | 0.20 | 8.00 | 0.19 |
|  | R3 | 9.00 | 0.15 | 8.00 | 0.20 | 8.00 | 0.16 | 7.00 | 0.15 |
| CTF | R1 | 12.00 | 0.14 | 13.00 | 0.18 | 10.00 | 0.15 | 11.00 | 0.14 |
|  | R2 | 11.00 | 0.16 | 12.00 | 0.21 | 11.00 | 0.17 | 10.00 | 0.16 |
|  | R3 | 12.00 | 0.15 | 12.00 | 0.20 | 10.00 | 0.16 | 11.00 | 0.15 |
| FTF | R1 | 12.00 | 0.20 | 11.00 | 0.25 | 10.00 | 0.21 | 8.00 | 0.20 |
|  | R2 | 11.00 | 0.16 | 12.00 | 0.21 | 9.00 | 0.17 | 9.00 | 0.16 |
|  | R3 | 11.00 | 0.12 | 11.00 | 0.15 | 8.00 | 0.13 | 8.00 | 0.12 |
|  | Grain yield (t/ha) | | | | | | | | |
|  |  | AWD 2022 | SD | AWD 2023 | SD | FL 2022 | SD | FL 2023 | SD |
| NTF | R1 | 4.31 | 0.12 | 3.88 | 0.14 | 3.84 | 0.12 | 3.63 | 0.15 |
|  | R2 | 4.13 | 0.19 | 4.31 | 0.21 | 3.83 | 0.17 | 3.42 | 0.23 |
|  | R3 | 3.79 | 0.15 | 4.17 | 0.17 | 3.56 | 0.14 | 3.54 | 0.19 |
| CTF | R1 | 5.29 | 0.14 | 5.25 | 0.16 | 4.54 | 0.13 | 4.90 | 0.18 |
|  | R2 | 5.27 | 0.16 | 5.08 | 0.18 | 5.10 | 0.15 | 5.20 | 0.20 |
|  | R3 | 5.07 | 0.15 | 5.28 | 0.17 | 5.29 | 0.14 | 4.90 | 0.19 |
| FTF | R1 | 5.11 | 0.19 | 4.88 | 0.22 | 4.32 | 0.18 | 4.46 | 0.24 |
|  | R2 | 5.36 | 0.16 | 5.19 | 0.18 | 4.71 | 0.15 | 4.28 | 0.20 |
|  | R3 | 5.21 | 0.11 | 5.09 | 0.13 | 4.44 | 0.11 | 4.61 | 0.14 |

**Supplementary table 7**. Rice plant physiological measurements and yield in all the experimental setups. Here, the table represents average data from the 12 experimental sites in triplicate; that is, R1, R2 and R3 all represent the average values of 12 sites.

**Python Coding for the global NTF, CTF, and FTF map projections-**

import pandas as pd

import plotly.graph_objects as go

# Load the data

df = pd.read_csv('no_tillage_co2_emissions.csv')

# Create a mapping of country names to ISO codes for choropleth

country_iso_map = {

'United States': 'USA', 'China': 'CHN', 'India': 'IND', 'Brazil': 'BRA',

'Russia': 'RUS', 'Australia': 'AUS', 'Canada': 'CAN', 'Argentina': 'ARG',

'Indonesia': 'IDN', 'Mexico': 'MEX', 'Turkey': 'TUR', 'Kazakhstan': 'KAZ',

'Iran': 'IRN', 'Ukraine': 'UKR', 'South Africa': 'ZAF', 'France': 'FRA',

'Germany': 'DEU', 'Thailand': 'THA', 'Spain': 'ESP', 'Poland': 'POL',

'Italy': 'ITA', 'United Kingdom': 'GBR', 'Myanmar': 'MMR', 'Pakistan': 'PAK',

'Bangladesh': 'BGD', 'Nigeria': 'NGA', 'Vietnam': 'VNM', 'Philippines': 'PHL',

'Ethiopia': 'ETH', 'Egypt': 'EGY', 'Romania': 'ROU', 'Czech Republic': 'CZE',

'Hungary': 'HUN', 'Belarus': 'BLR', 'Greece': 'GRC', 'Portugal': 'PRT',

'Azerbaijan': 'AZE', 'Serbia': 'SRB', 'Bulgaria': 'BGR', 'Austria': 'AUT',

'Morocco': 'MAR', 'Tunisia': 'TUN', 'Algeria': 'DZA', 'Libya': 'LBY',

'Sudan': 'SDN', 'Chad': 'TCD', 'Niger': 'NER', 'Mali': 'MLI',

'Burkina Faso': 'BFA', 'Ghana': 'GHA'

}

# Add ISO codes to dataframe

df['iso_code'] = df['Country'].map(country_iso_map)

# Create the choropleth map

fig = go.Figure(data=go.Choropleth(

locations=df['iso_code'],

z=df['CO2_Emissions_kg_ha_year'],

text=df['Country'],

colorscale=[

[0, '#90EE90'], # Light green for low emissions

[0.5, '#32CD32'], # Medium green

[1, '#006400'] # Dark green for high emissions

],

autocolorscale=False,

marker_line_color='darkgray',

marker_line_width=0.5,

colorbar=dict(

title="CO2 kg/ha/yr",

len=0.7

),

hovertemplate='<b>%{text}</b><br>' +

'CO2: %{z:.1f} kg/ha/yr<br>' +

'<extra></extra>'

))

fig.update_layout(

title='CO2 from No-Till Agriculture',

geo=dict(

showframe=False,

showcoastlines=True,

projection_type='equirectangular'

)

)

# Save the chart

fig.write_image('NTF_co2_emissions_map.png')

import pandas as pd

import plotly.express as px

# Load the data

df = pd.read_csv("furrow_tillage_co2_emissions.csv")

# Display basic info about the data

print("Data shape:", df.shape)

print("Columns:", df.columns.tolist())

print("Sample data:")

print(df.head())

print("\nCO2 emissions range:")

print(f"Min: {df['CO2_Emissions_kg_ha_year'].min():.2f}")

print(f"Max: {df['CO2_Emissions_kg_ha_year'].max():.2f}")

# Create choropleth map

fig = px.choropleth(

df,

locations="Country",

locationmode="country names",

color="CO2_Emissions_kg_ha_year",

hover_name="Country",

hover_data={"CO2_Emissions_kg_ha_year": ":.2f"},

color_continuous_scale="Oranges",

title="Global CO2 from Furrow Tillage"

)

# Update layout for better appearance

fig.update_layout(

coloraxis_colorbar=dict(

title="CO2 kg/ha/yr"

),

geo=dict(

showframe=False,

showcoastlines=True,

projection_type='equirectangular'

)

)

# Save the chart

fig.write_image("FTF_co2_map.png")

import pandas as pd

import plotly.express as px

import plotly.graph_objects as go

# Load the data

df = pd.read_csv("deep_tillage_co2_emissions.csv")

# Display the data structure to understand it

print("Data columns:", df.columns.tolist())

print("First few rows:")

print(df.head())

print("\nData shape:", df.shape)

print("\nCountry names sample:", df['Country'].unique()[:10])

# Create choropleth map

fig = px.choropleth(

df,

locations='Country',

color='CO2_Emissions_kg_ha_year',

locationmode='country names',

color_continuous_scale='Reds',

title='CO2 from Deep Tillage by Country',

labels={'CO2_Emissions_kg_ha_year': 'CO2 kg/ha/yr'}

)

# Update layout for better appearance

fig.update_layout(

coloraxis_colorbar=dict(

title="CO2 kg/ha/yr",

title_font_size=12

)

)

# Update geos for better country boundaries

fig.update_geos(

showframe=False,

showcoastlines=True,

projection_type='equirectangular'

)

# Save the chart

fig.write_image("CTF_co2_emissions_map.png", width=1200, height=600)
